# Psychosocial functioning in children with a congenital heart disease: attachment and emotion regulation strategies of children and parents as explanatory factors

**DOI:** 10.3389/fcvm.2025.1658513

**Published:** 2025-12-17

**Authors:** Saskia Mels, Katya De Groote, Kristof Vandekerckhove, Lien Goossens

**Affiliations:** 1 Department of Pediatrics, Pediatric Psychology, Ghent University Hospital, Ghent, Belgium; 2 Department of Pediatrics, Pediatric Cardiology, Ghent University Hospital, Ghent, Belgium; 3 Department of Developmental, Personality and Social Psychology, Faculty of Psychology and Educational Sciences, Ghent University, Ghent, Belgium

**Keywords:** emotion regulation, psychological adaptation, child psychology, congenital heart defects, parent–child relations, internalizing and externalizing disorders, family therapy

## Abstract

**Introduction:**

Children born with congenital heart disease (CHD) face increased risk of psychosocial difficulties. These may stem from challenges in parent–child relationships and related emotion regulation (ER) processes. This study examined whether insecure attachment and maladaptive parental emotion regulation strategies (ERS) are associated with psychological functioning in children with CHD. We also investigated whether these effects are associated through pathways involving children's own ER.

**Methods:**

In a sample of 218 families, with children between the ages 8 and 18 and their parents, participants completed questionnaires on attachment [Experiences in Close Relationships Scale–Revised Child (ECR-RC)], emotion regulation [Fragebogen zur erhebung der emotions regulation bei kindern und jugendlichen (FEEL-KJ/FEEL-E)], and psychosocial functioning [Strengths and Difficulties Questionnaire (SDQ)].

**Results:**

Results showed that insecure attachment to mothers, but not fathers, had direct effects on children's self-reported psychological difficulties, particularly internalizing symptoms. These effects were partially associated through pathways involving children's maladaptive ER. Mother-reported outcomes mirrored these findings, while father-reported outcomes primarily revealed associations via associative pathways. Parental maladaptive ER showed no significant effects based on child or mother reports but did show both direct effects and associations via associative pathways based on father reports.

**Discussion:**

These findings highlight the importance of fostering secure parent–child relationships and strengthening children's ER skills through targeted interventions to better support psychological wellbeing in children with congenital heart disease.

## Introduction

Congenital heart disease (CHD) is a structural abnormality of the heart's chambers, valves, or vessels (including the intrathoracic great vessels) that occurs in 0.8% of births. It varies in severity and outcomes (1, 2). This heterogeneity requires differentiation in medical treatments, including neonatal hospitalization, multiple medical interventions (early or later in life), surgical complexity, and lifelong follow-up. With improved medical care, life expectancy has increased (3).

Receiving a diagnosis of CHD, and undergoing corrective treatment, can be distressing for children and their parents and may affect children's psychosocial development. Episodes of care can involve loss of control over health and body. These include preventive clinic visits, acute care, medical procedures, and hospitalizations. They may also cause separation from parents and friends, and difficulties attending school or hobbies. Each of these factors can be emotionally challenging. Feelings of helplessness, fear and pain can contribute to stress and a pervasive sense of powerlessness in children (4, 5). The medical context may overwhelm children's capacity to regulate associated stress, potentially impairing emotional, social, and behavioral development (6, 7). Children with corrected CHD face increased risk of learning deficits, autism, and attention deficit hyperactivity disorder. They can also show emotional difficulties, including dysregulation, internalizing symptoms (e.g., anxiety, depression), and externalizing behaviors (e.g., aggression, hyperactivity) (8–11). By adolescence, they may develop psychopathology, including anxiety disorders, depression, or behavioral problems (12–14). Psychological outcomes in CHD vary with disease severity, with more complex conditions linked to more severe psychosocial problems. Parents often report greater emotional and behavioral difficulties than the youngsters themselves. This highlights the influence of disease severity and informant perspective, though underlying mechanisms remain poorly understood (15, 16).

Emotion regulation (ER) is assumed to play a role in the development of psychopathology. ER is an underlying mechanism that explains how and why childhood distress can lead to psychosocial dysfunction (17). ER refers to the ability to regulate emotional reactions (18). Emotion regulation strategies (ERS) are ways to control emotions, influencing their occurrence, timing, experience, and expression (19). ERS are often divided into two categories (20): adaptive (e.g., positive refocusing, acceptance) and maladaptive strategies (e.g., self-blame, rumination). ER begins early in life through behaviors and skills that modulate, inhibit, or enhance emotional responses. These skills become increasingly complex throughout development, shaped by intrinsic factors (e.g., temperament) and extrinsic influences (e.g., caregiver modeling) (21).

Children with corrected CHD may experience various emotions due to their complex medical condition and associated stressors (22). In this context, ER plays a crucial role in supporting their psychological adjustment, social functioning, and overall wellbeing (7). Understanding the underlying emotional mechanisms in children with corrected CHD is essential, as these influence how they cope with illness-related challenges. Furthermore, the familial context, particularly parental emotional responses, can significantly shape a child's emotional experiences and ER development. The tripartite model (23) describes how familial factors such as parent–child attachment and parental ERS influence children's emotional and behavioral outcomes. This model suggests familial factors affect children's maladjustment and wellbeing directly and via associative pathways through children's own ER. For a more detailed visual representation of these pathways, please refer to the conceptual model of ER development by Morris et al. (23).

Insecure attachment refers to the child's lack of confidence in the availability or responsiveness of the caregiver and has been associated with greater emotional vulnerability and reduced use of adaptive ER strategies (24). In CHD, repeated hospitalizations and parental separation may disrupt secure attachment development (11). Parents experience distress from (fetal) diagnosis through treatment and follow-up. Stress can impair parental behavior and parent–child relationships (25–27). This affects parents' ability to respond sensitively to cues, in attachment-forming interactions (11).

Parental ERS refer to the cognitive and behavioral strategies parents use to manage their own emotional responses. These strategies range from adaptive (e.g., positive reframing) to maladaptive (e.g., catastrophizing). They shape the home emotional climate and parental responses to children's needs (23, 28). Children internalize and model parental ERS, making parental ER a significant developmental factor.

Although earlier research has shown elevated risks for psychological distress and maladjustment in children with corrected CHD, few studies have investigated how child insecure attachment and maladaptive parental ERS influence psychosocial outcomes in this group. Also, the potential role of associative pathways such as children's own maladaptive ERS has received limited empirical attention. Addressing this gap, the current study integrates theoretical and empirical perspectives to examine these interrelated mechanisms within families.

This explorative, observational cross-sectional study of children with corrected CHD examines, based on child-, mother-, or father-reported outcomes, (1) whether insecure attachment and (2) maladaptive parental ERS are directly associated with the children's psychosocial functioning and whether this is associated through a pathway with children's maladaptive ERS.

## Methods

### Participants and procedure

The hospital's Ethics Committee (BC-07779) granted approval of the protocol on 18 August 2020.

Pediatric patients aged 8–18 with corrected CHD were recruited from the pediatric CHD database of the university hospital. Only children with corrected CHD (defects treated through percutaneous and/or surgical interventions) were included. CHD severity was classified according to the Bethesda criteria (29), in three categories (see Table 1): simple (e.g., atrial septal defects), moderate (e.g., Ebstein anomaly), and complex CHD (e.g., hypoplastic left heart syndrome). Families with insufficient Dutch language knowledge were excluded. Children with complex non-cardiac pathology (e.g., cerebral palsy, metabolic disorders), genetic abnormalities, or intellectual disabilities were also excluded. The lower age limit was 8 years, when children can reliably complete standardized questionnaires independently. The upper limit of 18 years reflects the boundary of pediatric care in our center.

**Table 1:** Clinical and demographic data of children (8–18 years) with CHD (*N* = 218).

Variable	Category	Value (Mean ± SD or N (%))	
		Mean	(*±*SD)
Age (years)		12.21	(±2.86)
		*N*	(%)
Biological sex			
	Male	118	(54.1)
Education			
	Primary school	93	(42.7)
	Special needs education	11	(5.0)
	ASO—secondary school (academic track; i.e., preparing for college or university)	72	(33.0)
	TSO—secondary school (technical track; i.e., preparing for technical proficiencies)	23	(10.6)
	KSO—secondary school (arts track; i.e., preparing for arts proficiencies)	1	(0.5)
	BSO—secondary school (craft track; i.e., preparing for craft proficiencies)	11	(5.0)
	Other (e.g., going to school in other country)	7	(3.2)
Family composition			
	Living together with both parents (intact family)	152	(69.7)
	Co-parenting (after divorce)	17	(7.8)
	Living with their mother	5	(2.3)
	Living with their mother and new partner	8	(3.7)
	Living with their father and new partner	2	(0.9)
	Living with their grandparents	2	(0.9)
	Information was missing	32	(14.7)
Birth order			
	Oldest child	79	(36.2)
	Second child	75	(34.4)
	Third child	23	(10.6)
	Fourth child	6	(2.8)
	Fifth child	1	(0.5)
	Sixth child	2	(0.9)
	Information was missing	32	(14.7)
Heart defect in child^a^			
Simple complexity		73	(33.5)
	Atrial septal defects (ASD I, ASD II, or sinus venosus ASD)	39	(17.9)
	Ventricular septal defect (VSD)	26	(11.9)
	Aortic valve stenosis	8	(3.7)
Moderate complexity		87	(39.9)
	Atrioventricular septal defect (AVSD)	8	(3.7)
	Coarctation of the Aorta (CoA and CoA with VSD)	33	(15.1)
	d-Transposition of the Great Arteries (d-TGA)	20	(9.2)
	Pulmonary valve stenosis	10	(4.6)
	Coronary abnormality	3	(1.4)
	Ebstein anomaly	4	(1.8)
	Total Anomalous Pulmonary Venous Return (TAPVU)	2	(0.9)
	Other cardial abnormalities	7	(3.2)
Severely complex		58	(26.6)
	Mitralvalve stenosis	5	(2.3)
	Double-outlet right ventricle (DORV)	3	(1.4)
	Truncus arteroisus	2	(0.9)
	Pulmonary atresia with intact septum	5	(2.3)
	Tetralogy of Fallot (TOF and TOF with MAPCA's)	22	(10.1)
	Hypoplastic left heart syndrome (HLHS)	15	(6.9)
	Aortic valve stenosis	5	(2.3)
	Other cardial abnormalities	1	(0.4)
Timing diagnosis			
	Prenatal	29	(13.4)
	Postnatal	188	(86.2)
	Missing	1	(0.4)
Admission to NICU after birth	Yes	114	(52.3)

*N*, number; SD, standard deviation; NICU, neonatal intensive care unit.

a Heart defect severity: in the absence of a comprehensive assessment of disease severity in children with CHD, the child cardiologists generated a classification system, similar to the one used in adults with CHD (Task Force 1 of the 32nd Bethesda conference of the American College of Cardiology, 2001). The Bethesda disease complexity classification categorizes patients into three groups: simple, moderate, and severely complex congenital heart defects, based solely on the anatomic complexity.

Invitations were distributed using anonymous family codes to ensure confidentiality; the researcher had no access to identifiable data. Study information was sent to 592 families; 86 could not be reached due to outdated contact details. Out of the remaining families, 218 agreed to take part. Participation was voluntary, and no incentives were offered. Families were informed that full triad participation was not needed, and that data from individual participants were equally valued. Each respondent (child, mother, father) received age-appropriate study information, an informed consent (or assent) document, and a unique login code to a secure website. Respondents within the same family received linked unique codes (e.g., dsa-423c = child, dsa-423 m = mother, and dsa-423f = father), enabling structured and anonymous pairing of informant data. Data collection ran from 17 November 2020, to 9 November 2021. Children and parent(s) completed validated and standardized Dutch questionnaires online. Parents also reported on child and family sociodemographics, while medical data were obtained from a cardiologist-completed information sheet. Missing data were minimal, since the survey required responses to proceed but included a “prefer not to answer” option. Missing values were handled in accordance with the established questionnaire guidelines.

### Measures

Unless otherwise noted, measures were based on child self-report. Parent-report data appear in the Results and in Supplementary Materials for clarity.

#### Experiences in close relationships scale-revised child version

To assess insecure attachment, children aged 8–18 completed the Dutch self-report Experiences in close relationships scale-revised child (ECR-RC) questionnaire (30). The questionnaire includes 12 items with a stable two-factor-structure: attachment-related anxiety (preoccupation with social support, jealousy, fear, and vigilance concerning abandonment and rejection) and avoidance (avoidance of intimacy, discomfort with closeness, and self-reliance). Both subscales (“Attachment Anxiety” and “Attachment Avoidance”) prove high internal consistency, reliability, and validity in children (8–12 years) and adolescents (13–18 years). Original studies have demonstrated good psychometric properties, with Cronbach's alphas ranging from 0.89 to 0.92 for Anxiety (maternal and paternal ratings) and from 0.93 to 0.94 for Avoidance (maternal and paternal ratings) (30–32). Children rated their responses toward each parent on a 7-point Likert scale, ranging from 1 (strongly disagree) to 7 (strongly agree). Scores were obtained for “Attachment Anxiety” (e.g., “I worry that my father/mother does not really love me”) and “Attachment Avoidance” (e.g., “I prefer not to tell my father/mother how I feel deep down”). A higher score shows a more insecure attachment pattern of this type. In this study, the internal consistency for the child's self-reported “Attachment Anxiety toward Mother” was 0.84 and “Attachment Anxiety toward Father” was 0.94, while “Attachment Avoidance toward Mother” was 0.88 and “Attachment Avoidance toward Father” was 0.87. Beyond its application in prior research by the current authors (33), this instrument's use within CHD populations is, to our knowledge, unprecedented in the published literature.

#### Strengths and difficulties questionnaire

The psychosocial functioning of the child was assessed using the standardized Dutch Strengths and difficulties questionnaire (SDQ) (34–36) through self- and parent-reports. While child self-reports are available for children between the ages 11 and 18 years, parents can complete reports for children between the ages 4 and 18 years. The questionnaire includes 20 items capturing four difficulty domains: hyperactivity/inattention (e.g., “Restless, overactive, cannot stay still for long”), emotional (e.g., “Many fears, easily scared”), conduct (e.g., “Often has temper tantrums or hot tempers”), and peer (e.g., “Picked or bullied by others”) problems. The conduct and hyperactivity/inattention items form the externalizing scale, while the emotional and peer items form the internalizing scale. Responses are rated on a 3-point scale (0 = “not true,” 1 = “somewhat true,” and 2 = “certainly true”), with higher scores showing a higher level of difficulty. Evidence from Dutch studies supports the validity and internal consistency of the SDQ, which is validated for children and adolescents aged 4–18 years. Original studies report Cronbach's alphas between 0.70 and 0.85 for the externalizing scale and between 0.70 and 0.81 for the internalizing scale, varying depending on the respondent (35, 37, 38). In this study, the Cronbach's alphas for the externalizing scale were 0.81 (mother reported), 0.79 (father reported), and 0.78 (child reported), and for the internalizing scales, they were 0.81 (mother reported), 0.80 (father reported), and 0.72 (child reported). Although less common in CHD research than the Youth Self-Report (YSR; child self-report) or Child Behavior Checklist (CBCL; parent report) (39), the SDQ has been applied in CHD populations (6, 33) and is valued for its brevity.

#### Fragebogen zur erhebung der emotionsregulation bei kindern und jugendlichen

Children's ERS (8–18 years) were measured using the Dutch self-report version of the Fragebogen zur erhebung der emotionsregulation bei kindern und jugendlichen (FEEL-KJ) (40). Ninety items measure 15 different ERS, each strategy assessed independently using two items for anger, sadness, and anxiety. Seven strategies are classified as Adaptive ER and five as Maladaptive ER. Three remaining strategies (social support, expression, and emotional control) could not be classified. Children respond on a 5-point Likert scale, ranging from 1 (“almost never”) to 5 (“almost always”). Each score on the scales shows the child's use of ERS; higher scores show more use of those ERS. Psychometric evaluation revealed a good internal consistency, good test–retest reliability, and consistent associations with anxiety and depression measures. The instrument shows strong psychometric properties, with original Cronbach's alphas between 0.81 and 0.95 across strategies (41). In this study, only “Maladaptive ER” was used, including five strategies: (1) Giving Up (e.g., “I don’t want to do anything”), (2) Aggressive Actions (e.g., “I get into a quarrel with others”), (3) Withdrawal (e.g., “I don’t want to see anyone”), (4) Self-Devaluation (e.g., “I blame myself”), and (5) Rumination (e.g., “I cannot get it out of my head”). In our study, Cronbach's alpha for the child's self-reported “Maladaptive ER” is 0.85. Adaptive ERS are being analyzed in a complementary study with different research questions and will be reported separately. The present study focuses solely on maladaptive ERS pathways.

#### Fragebogen zur erhebung der emotionsregulation bei erwachsenen

To gain insight into parental ERS, the Dutch version of the Fragebogen zur erhebung der emotions regulation bei erwachsenen (FEEL-E), similar to the FEEL-KJ, was administered, comprising 72 items measuring only 12 strategies related to anger, sadness, and anxiety (42). Six strategies are classified as “Adaptive ER” and six as “Maladaptive ER.” Parents respond on a 5-point Likert scale, ranging from 1 (“almost never”) to 5 (“almost always”). In this study, only parental “Maladaptive ER” was analyzed: (1) Giving Up (e.g., “I can’t do anything about my fear anyway”), (2) Withdrawal (e.g., “I withdraw”), (3) Self-Devaluation (e.g., “I think it's my own problem”), (4) Rumination (e.g., “I always wonder why I am sad”), and two additional factors: (5) “Negative Thinking” (e.g., “I think what I’ve been through is worse than what other people are going through”) and (6) “Blaming Others” (e.g., “I think others are responsible for what happened”). The “Maladaptive ER” scale includes general dispositions to maladaptively cope with negative emotions. The FEEL-E is validated for use in adults and shows excellent psychometric properties with original Cronbach's alphas for the Maladaptive scale ranging from 0.78 to 0.93 (42). In our study, Cronbach's alpha for parental “Maladaptive ER” is 0.92 (mother) and 0.93 (father). Parental adaptive ERS will be reported elsewhere; this study focuses solely on maladaptive ERS.

### Statistical analysis

An *a priori* power analysis, conducted with G*Power 3.1.9.2 (43), indicated that approximately 71–116 families were needed to detect a medium-sized indirect effect in a simple associative pathway model (ab = 0.15, *α* = 0.05, power = 0.80–0.90). To account for multiple informants and complex pathways, we deliberately oversampled.

The total number of unique participating families was 218. Research questions were examined using a multi-informant approach. This included child self-reported (*N* = 107), mother-reported (*N* = 192), and father-reported (*N* = 153) outcome measures. Supplementary Table S1 summarizes participant constellations and response rates. Across instruments, missing data were minimal. The median number of missing items was 0 for the ECR-RC (toward mother and toward father), 0 for the SDQ (child reports, mother, and father outcome reports), 0 for the FEEL-KJ (child and mother outcome reports) and 1 for the FEEL-KJ (father outcome reports), and 0 for the FEEL-E (mother and father reports).

Statistical analysis was performed using SPSS (version 28; IBM, SPSS Inc., Chicago, IL, USA). In preliminary analyses, we systematically explored potential covariates to identify variables for inclusion in final models. These included sociodemographic factors (family composition, household size, child's nationality, child's education level, and birth order) as well as clinical characteristics [child's age, child's biological sex, CHD type and severity, timing of diagnosis, neonatal intensive care unit (NICU) admission, and number of surgeries]. Associations between continuous child variables (age, number of surgeries), family variables (household size), and outcome variables were calculated using Pearson's correlations. Multivariate analysis of variance (MANOVA) was used to examine differences in outcome variables based on categorical child variables (biological sex, birth order, nationality, type/severity of heart defect, type of surgery, timing of diagnosis, or NICU admission), family variables (composition), and outcome variables. This exploration of covariates followed standard practice to support model parsimony and avoid overfitting.

We used the PROCESS macro (Model 4) (44), to explore associative pathways as an exploratory tool rather than for causal mediation. To control for Type I errors, Bonferroni-adjusted alpha thresholds were applied: *α* = 0.013 for analyses related to child attachment and *α* = 0.025 for analyses related to parental maladaptive ERS. Significance criteria for indirect effects were evaluated using bias-corrected bootstrap analyses with 5,000 samples and 95% confidence intervals (CIs). Effects were considered significant when the CI did not include zero (44). Effects for which the CI narrowly included or narrowly excluded zero are described as “borderline” and interpreted with caution. First, to examine the association between the child's attachment insecurity and the child's psychological functioning and whether this is possibly associated through associative pathways with the child's maladaptive ER, 2 × 4 associative pathway models were run (“Attachment Anxiety” or “Attachment Avoidance” toward Mother/Father as predictor and internalization/externalization problems as outcome).

Second, to examine the association between parents’ maladaptive ERS and the child's psychological functioning and whether this is associated through associative pathways with the child's maladaptive ER, 2 × 2 associative pathway models were run (mother's/father's maladaptive ERS as predictors and internalization/externalization problems as outcomes). Unstandardized coefficients were reported, and effect sizes for indirect effects were reported as completely standardized indirect effect (CSIE) (45).

## Results

### Sample characteristics

Children with corrected CHD (*N* = 118 boys, *N* = 100 girls) were the study subjects. In two families, the grandmother and grandfather took part instead of the biological parents. Two respondents turned 18 years old during the study participation.

The age at first CHD surgery ranged from day 1 to the age of 14.3 years. Ninety-one children had at least one percutaneous procedure, and 186 had at least one surgical intervention. Thirty-two patients only underwent percutaneous treatment. Given unequal group sizes (32 percutaneous only vs. 186 with at least one surgical intervention), we tested whether these groups differed on key variables based on clinical expected psychological differences. Self-reports showed no statistical differences between groups on key variables: child’s Maladaptive ERS or Maladaptive ERS Child (M.ERS-C) [F(1,93) = 1.203, *p* = 0.276], Attachment Anxiety toward Mother [F(1,109) = 0.086, *p* = .770], Attachment Anxiety toward Father [F(1,100) = 2.071, *p* = .153], Attachment Avoidance toward Mother [F(1,109) = 3.287, *p* = .073], or Attachment Avoidance toward Father [F(1,100) = 1.712, *p* = .194]. Outcome measures “Internalizing/Externalizing problems” as reported by the child were not significantly related to the type of surgery, respectively, F(1,107) = 0.639, *p* = 0.426, and F(1,107) = 1.179, *p* = 0.280. Based on mother and father outcome reports, the same results were found. Therefore, these 32 children, who only underwent percutaneous treatment, were not excluded from the analyses. Other clinical and demographic data on the children with CHD (*N* = 218) can be found in Table 1.

In total, 345 parents (*N* = 192 mothers, *N* = 153 fathers) participated in this study; 55.7% were females (188 biological mothers, three stepmothers, and one grandmother) and 44.3% were males (145 biological fathers, seven stepfathers, and one grandfather). Other demographic data on parents can be found in Table 2.

**Table 2:** Demographic data of the parents of children with CHD.

Variable (and category)	Value (N (%))	
	Male (*N* = 153)	Female (*N* = 192)
	*N* (%)	*N* (%)
Highest education level obtained		
Primary school	0 (0.0)	1 (0.5)
Secondary school—incomplete	3 (2.0)	8 (4.2)
Secondary school—completed	51 (33.3)	43 (22.4)
College (short type)	42 (27.5)	65 (33.9)
College (long type)	22 (14.4)	36 (18.8)
University	33 (21.6)	38 (19.8)
Another type of highest education level	2 (1.3)	1 (0.5)
Occupation		
Worker	23 (15.0)	20 (10.4)
Attendant	71 (46.4)	95 (49.5)
Self-employed (own business)	29 (19.0)	17 (8.9)
Self-employed (in another's business)	3 (2.0)	4 (2.1)
Functionary	23 (15.0)	41 (21.4)
Housework	1 (0.7)	4 (2.1)
Student	0 (0.0)	2 (1.0)
Disabled	1 (0.7)	6 (3.1)
Retired	1 (0.7)	1 (0.5)
Unemployed	0 (0.0)	2 (1.0)
Another type of employment	1 (0.7)	0 (0.0)

*N* = number.

### Preliminary analysis

Prior to the analyses, descriptive statistics and correlations among the child study variables were examined to gain insight into their interrelationships. Descriptive statistics and correlations among the child study variables can be found in Table 3. All variables were normally distributed, mean ± SD were used to describe the parameters. Significant correlations between variables were seen in the expected directions. The same were conducted using parent-reported outcomes (see Supplementary Material Tables S2, S3).

**Table 3:** Descriptives and Pearson correlations among child study variables (*N* = 107).

Variable	*M*	(SD)	1	2	3	4	5	6	7	8	9
1. Att. Anx. M	9.36	(4.79)	–								
2. Att. Av. M	19.31	(8.99)	0.348**	–							
3. Att. Anx. F	9.36	(5.44)	0.721**	0.190	–						
4. Att. Av. F	21.71	(8.66)	0.244*	0.630**	0.318**	–					
5. Mal. ERS M	92.60	(18.48)	0.118	0.240*	0.164	0.289**	–				
6. Mal. ERS F	89.25	(16.74)	0.153	0.226	0.201	0.157	0.427**	–			
7. Mal. ERS C	82.75	(13.81)	0.372**	0.266*	0.301**	0.343**	0.146	0.184	–		
8. Int.	5.49	(3.42)	0.305**	0.363**	0.410**	0.377**	0.172	0.201	0.560**	–	
9. Ext.	6.30	(3.63)	0.380**	0.385**	0.221*	0.156	0.099	0.193	0.064	0.167	–

Att. Anx. M, Attachment Anxiety toward Mother; Att. Av. M, Attachment Avoidance toward Mother; Att. Anx. F, Attachment Anxiety toward Father; Att. Av. F, Attachment Avoidance toward Father; Mal. ERS M (tot), Mother's maladaptive emotion regulation strategies Maladaptive ERS Mother (total); Mal. ERS F, Father's maladaptive emotion regulation strategies or Maladaptive ERS Father (total); Mal. ERS C, Child's maladaptive emotion regulation strategies or Maladaptive ERS Child (total); Int., Internalizing problems (child self-reports); Ext., Externalizing problems (child self-reports); M, mean; SD, standard deviation.

* *p* < .05 (two-tailed).

** *p* < .01 (two-tailed).

In preliminary analyses, we examined a broad set of potential covariates. The externalizing outcome was unrelated to the child's biological sex, education, family composition, birth order, nationality, age (*r * =  –0.011, *p* = 0.913), type of CHD, severity, diagnosis timing, NICU admission, household size, or number of surgeries (all *p* > 0.17). Consequently, no covariates were included in associative pathways for externalizing outcomes.

The internalizing outcome correlated significantly with the child's biological sex [F(1,107) = 13.68, *p* = 0.001] and age (*r* = .235, *p* = 0.015), and thus these were included as covariates in associative pathway models. All other variables (child's education, family composition, birth order, nationality, type and severity of CHD, timing of diagnosis, NICU admission, household size, and number of surgeries) were non-significant (*p* > .14) and therefore not retained as covariates.

Similar preliminary and associative pathway analyses were performed for mother and father outcome reports (see Supplementary Material S4–S9).

### Insecure attachment and internalizing problems, associated through associative pathways involving child's maladaptive ERS

#### Examining Attachment Anxiety toward Mother

**Figure 1:**
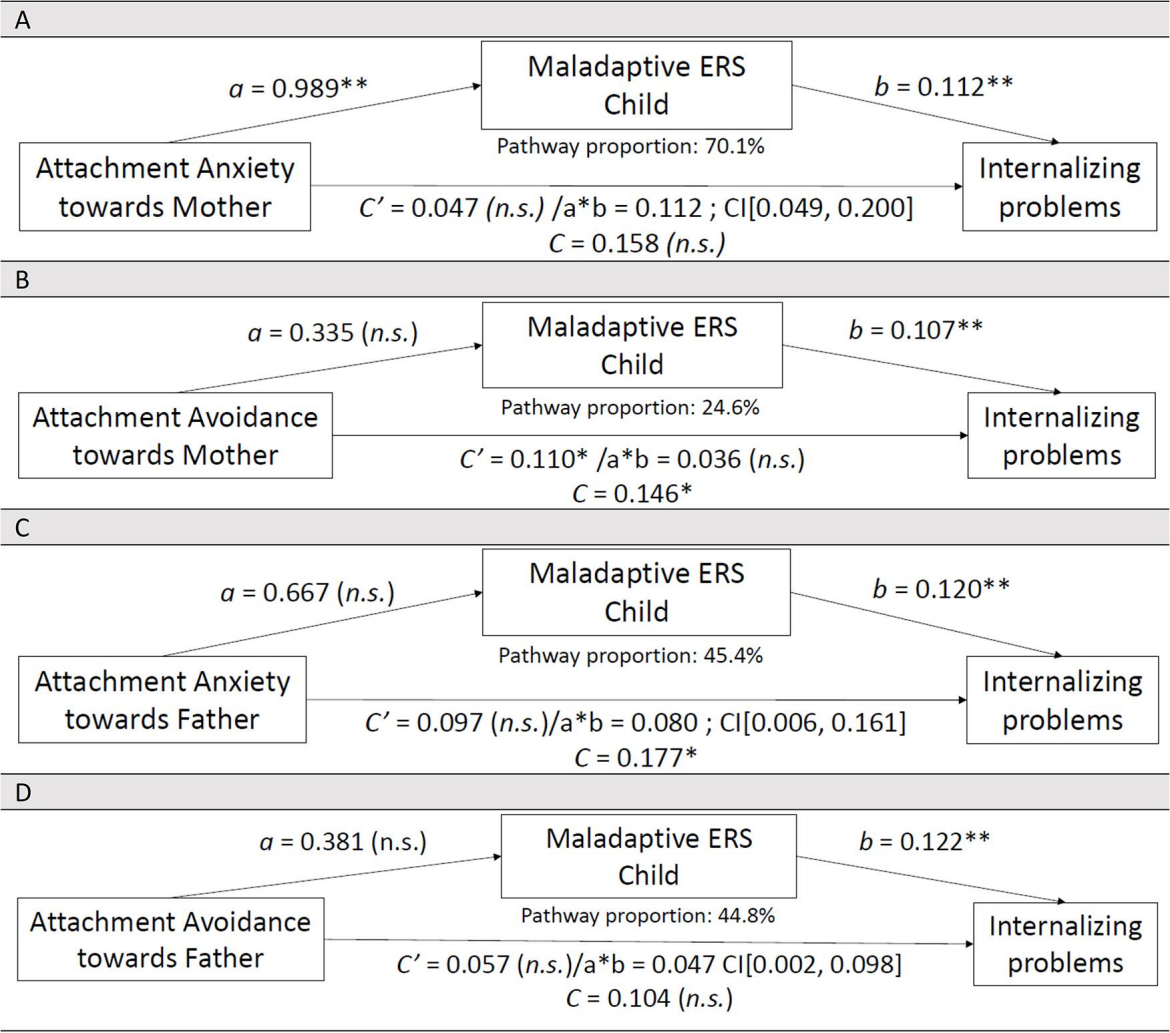
Associations between **(A)** Attachment Anxiety toward Mother (*N* = 90), or **(B)** Attachment Avoidance toward Mother (*N* = 91), or **(C)** Attachment Anxiety toward Father (*N* = 89), or **(D)** Attachment Avoidance toward Father (*N* = 89), Maladaptive ERS Child and Internalizing problems in 8- to 18-year-old children with CHD (child self-reports). Controlled for the child's age and the child's biological sex in these associative pathway analyses. n.s., not significant; Bonferroni correction (*p*-value of 0.05 divided by 4 = 0.013): *p < 0.013, **p < 0.001. *N* refers to the effective sample size per associative pathway model.

Figure 1A shows that the direct relationship between Attachment Anxiety toward Mother and Internalizing problems was not significant (SE = 0.071, *t* = 0.669, *p* = 0.505). Relationship between Attachment Anxiety toward Mother and M.ERS-C was significant (SE = .283, *t* = 3.500, *p* = 0.001). Relationship between M.ERS-C and Internalizing problems was significant (SE = .026, *t* = 4.442, *p* = 0.000). Examining the CI, an association via an associative pathway was suggested between Attachment Anxiety toward Mother on Internalizing problems through M.ERS-C (SE = 0.038, 95% CI narrowly excluded zero); this is considered borderline and must be interpreted with caution. Based on mother and father outcome reports, similar associations via associative pathways were observed. An overview of main associations and those associated through associative pathways involving the child's ERS, including unstandardized coefficients and CSIE values, is presented in Table 4.

**Table 4:** Overview of direct associations and associations through associative pathways involving the child's maladaptive ERS (child reports).

Predictor	Outcome	Model N	B	SE (ab)	95% CI	CSIE	Interpretation (child reports)	Note (parent outcome reports)
Att. Anxiety M	Int.	90	0.112	0.038	[0.049, 0.200]* CI excludes zero	0.048 (small)	Figure 1A: Sign. indirect effect; no direct effect	Replicated in mother/father reports
Att. Avoidance M	Int.	91	0.036	0.0192	[−0.002, 0.075]	0.015 (very small)	Figure 1B: No sign. indirect effect; direct effect sign.	Mother reports: no direct or indirect effect—Father reports: sign. indirect effect
Att. Anxiety F	Int.	89	0.080	0.0394	[0.006, 0.161]* CI excludes zero	0,031 (small)	Figure 1C: Sign. indirect effect; no direct effect	Borderline, replicated in mother/father reports
Att. Avoidance F	Int.	89	0.047	0.0243	[0.002, 0.098]* CI excludes zero	0.018 (small)	Figure 1D: Sign. indirect effect; no direct effect	Borderline, replicated in mother/father reports
Att. Anxiety M	Ext.	90	−0.024	0.0330	[−0.098, 0.033]	−0.009 (very small)	Figure 2A: No indirect effect; direct effect sign.	Mother reports: sign. direct effect—Father reports: no direct or indirect effect
Att. Avoidance M	Ext.	91	−0.004	0.0093	[−0.024, 0.014]	−0.002 (very small)	Figure 2B: No indirect effect; direct effect sign.	No mother/father report effects
Att. Anxiety F	Ext.	89	0.001	0.0222	[−0.045, 0.045]	0.0002 (very small)	Figure 2C: No sign. effects	Replicated in mother/father reports
Att. Avoidance F	Ext.	89	−0.000	0.0151	[−0.030, 0.032]	−0.00008 (very small)	Figure 2D: No indirect effect; no direct effect	Mother reports: sign. indirect effect—Father reports: no direct or indirect effect
Mal. ERS M	Int.	80	0.013	0.0105	[−0.006, 0.035]	0.0056 (very small)	Figure 3A: No indirect effect	Mother reports: no sign. effects
Mal. ERS F	Int.	67	0.018	0.0104	[−0.002, 0.040]	0.0079 (very small)	Figure 3B: No indirect effect	Father reports: both direct and indirect effects sign.
Mal. ERS M	Ext.	80	−0.000	0.0037	[−0.009, 0.008]	−0.00009 (negligible)	Figure 3C: No sign. effects	Mother reports: no sign. effects
Mal. ERS F	Ext.	67	0.002	0.0040	[−0.007, 0.010]	0.0006 (negligible)	Figure 3D: No indirect effect; no direct effect	Father reports: direct effect sign.

**Figure 2:**
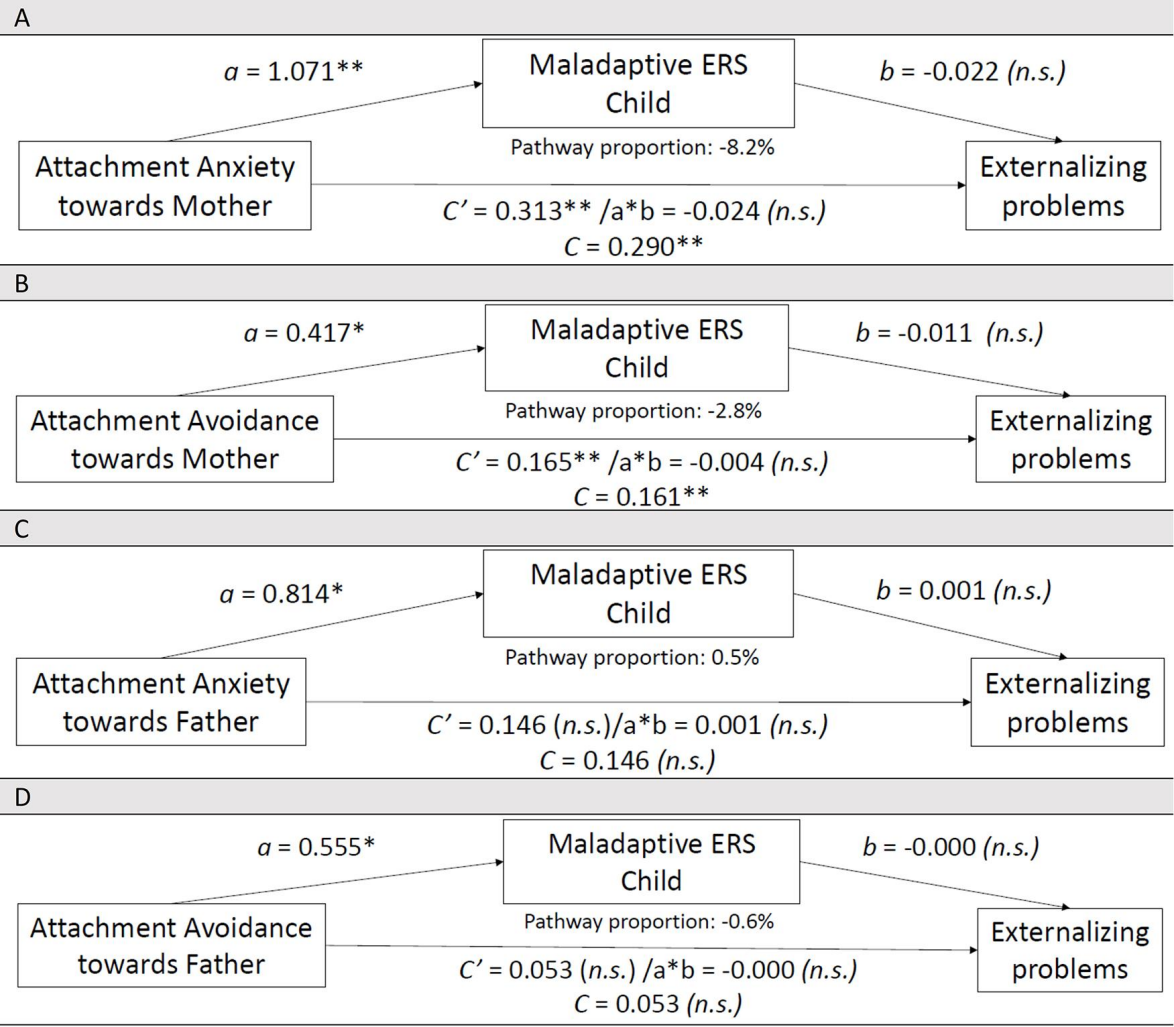
Associations between **(A)** Attachment Anxiety toward Mother (*N* = 90), or **(B)** Attachment Avoidance toward Mother (*N* = 91), or **(C)** Attachment Anxiety toward Father (*N* = 89), or **(D)** Attachment Avoidance toward Father (*N* = 89), Maladaptive ERS Child and Externalizing problems in 8- to 18-year-old children with CHD (child self-reports). Controlled for the child's age and the child's biological sex in these associative pathway analyses. n.s., not significant; Bonferroni correction (*p*-value of 0.05 divided by 4 = 0.013): *p < 0.013, **p < 0.001. *N* refers to the effective sample size per associative pathway model.

**Figure 3:**
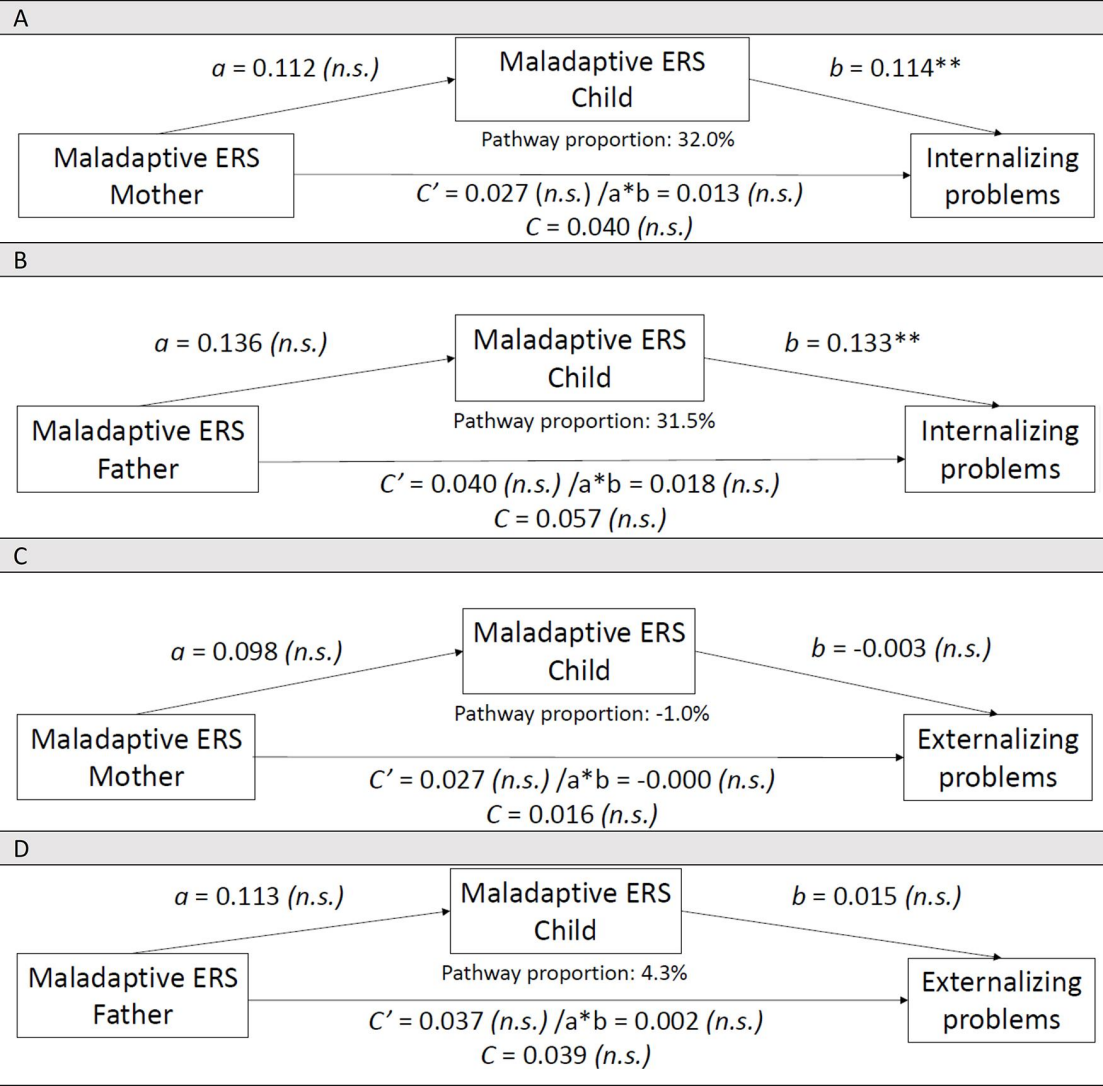
Associations between **(A)** Mother's maladaptive emotion regulation strategies (*N* = 80) or **(B)** Father's maladaptive emotion regulation strategies (*N* = 67), Maladaptive ERS Child and Internalizing problems in 8- to 17-year-old children with CHD (self-reports) associations between **(C)** Mother's maladaptive emotion regulation strategies (*N* = 80) or **(D)** Father's maladaptive emotion regulation strategies (*N* = 67), Maladaptive ERS Child and Externalizing problems in 8- to 18-year-old children with CHD (child self-reports). Controlled for the child's biological sex in these associative pathway analyses when "Internalizing problems" was the outcome variable. n.s., not significant; Bonferroni correction (*p*-value of 0.05 divided by 2 = 0.025): *p ≤ 0.025, **p ≤ 0.001. *N* refers to the effective sample size per associative pathway model.

ERS, emotion regulation strategies; Model N, sample size per model (child reports); Att. Anxiety (M), Attachment Anxiety toward Mother; Att. Avoidance (M), Attachment Avoidance toward Mother; Att. Anxiety F), Attachment Anxiety toward Father; Att. Avoidance F), Attachment Avoidance toward Father; Int., internalizing problems (child self-reports); Ext., externalizing problems (child self-reports); Mal. ERS M (tot), Mother's maladaptive emotion regulation strategies or Maladaptive ERS Mother (Total); Mal. ERS F), Father's maladaptive emotion regulation strategies or Maladaptive ERS Father (Total); SE (ab), standardized effect (ab); CI, confidence interval; CSIE, completely standardized indirect effect; Sign., significant (after Bonferroni correction (see also Figures 1–3).

Coefficients reported are unstandardized (B). Effect sizes are reported as completely standardized indirect effects [CSIE; Preacher and Kelley (45)]. CIs represent 95% confidence intervals.

* Associative pathway is significant (CI excludes zero). Effects with borderline CIs (close to zero) should be interpreted cautiously. Similar analyses were performed for mother and father outcome reports (see Supplementary Material S4–S9).

#### Examining Attachment Avoidance toward Mother

Figure 1B shows a significant direct relationship between Attachment Avoidance toward Mother and Internalizing problems (SE = 0.034, *t* = 3.250, *p* = 0.002). Relationship between Attachment Avoidance toward Mother and M.ERS-C was not significant (SE = 0.161, *t* = 2.077, *p* = 0.041). Relationship between M.ERS-C and Internalizing problems was significant (SE = 0.022, *t* = 4.883, *p* = 0.000). Examining the CI, no significant associative pathway was supported between Attachment Avoidance toward Mother on Internalizing problems via M.ERS-C (SE = 0.019, 95% CI −0.002,0.075); this is considered borderline and must be interpreted with caution. Based on parent outcome reports, no significant direct effects were found. Mother outcome reports found no significant associative pathway; however, for father outcome reports an association via an associative pathway was supported.

#### Examining Attachment Anxiety toward Father

Figure 1C shows no significant direct relationship between Attachment Anxiety toward Father and Internalizing problems (SE = 0.057, *t* = 1.693, *p* = 0.094). Relationship between Attachment Anxiety toward Father and M.ERS-C was not significant (SE = 0.275, *t* = 2.425, *p* = 0.017). Relationship between M.ERS-C and Internalizing problems was significant (SE = 0.022, *t* = 5.532, *p* = 0.000). Examining the CI, an association via an associative pathway was suggested between Attachment Anxiety toward Father on Internalizing problems through M.ERS-C (SE = 0.039, 95% CI narrowly above zero); this is borderline and must be interpreted cautiously. Based on parent outcome reports, same (in)direct associations were found.

#### Examining Attachment Avoidance toward Father

Figure 1D shows no significant direct relationship between Attachment Avoidance toward Father and Internalizing problems (SE = 0.037, *t* = 1.544, *p* = 0.126). Relationship between Attachment Avoidance toward Father and M.ERS-C was not significant (SE = 0.174, *t* = 2.191, *p* = 0.031). Relationship between M.ERS-C and Internalizing problems was significant (SE = 0.023, *t* = 5.414, *p* = 0.000). Examining the CI, an association via an associative pathway was suggested between Attachment Avoidance toward Father on Internalizing problems through M.ERS-C (SE = 0.024, 95% CI narrowly above zero); this is borderline and must be interpreted cautiously. Based on parent outcome reports, same (in)direct effects were found.

### Insecure attachment and externalizing problems, associated through associative pathways involving child's maladaptive ERS

#### Examining Attachment Anxiety toward Mother

Figure 2A shows the significant direct relationship between Attachment Anxiety toward Mother and Externalizing problems (SE = 0.083, *t* = 3.801, *p* = 0.000). Relationship between Attachment Anxiety toward Mother and M.ERS-C was significant (SE = 0.288, *t* = 3.712, *p* = 0.000). Relationship between M.ERS-C and Externalizing problems was not significant (SE = 0.028, *t* = −0.782, *p* = 0.436). Examining the CI, no significant associative pathway was supported between Attachment Anxiety toward Mother on Externalizing problems via M.ERS-C (SE = 0.033, 95% CI −0.0098, 0.033); this is considered borderline and must be interpreted with caution. Based on mother and father outcome reports, only for mother outcome reports the direct effect was significant. No significant associations via associative pathways were found.

#### Examining Attachment Avoidance toward Mother

Figure 2B shows the significant direct relationship between Attachment Avoidance toward Mother and Externalizing problems (SE = 0.041, *t* = 4.030, *p* = 0.000). Relationship between Attachment Avoidance toward Mother and M.ERS-C was significant (SE = 0.165, *t* = 2.523, *p* = 0.013). Relationship between M.ERS-C and Externalizing problems was not significant (SE = 0.026, *t* = −1.423, *p* = 0.674). Examining the CI, no significant associative pathway was supported between Attachment Avoidance toward Mother on Externalizing problems via M.ERS-C (SE = 0.009, 95% CI −0.024, 0.014). Based on parent outcome reports, no significant (in)direct associations were found.

#### Examining Attachment Anxiety toward Father

Figure 2C shows the direct relationship between Attachment Anxiety toward Father and Externalizing problems was not significant (SE = 0.076, *t* = 1.911, *p* = 0.059). Relationship between Attachment Anxiety toward Father and M.ERS-C was significant (SE = 0.281, *t* = 2.896, *p* = 0.005). Relationship between M.ERS-C and Externalizing problems was not significant (SE = 0.028, *t* = 0.030, *p* = 0.976). Examining the CI, no significant associative pathway was supported between Attachment Anxiety toward Father on Externalizing problems via M.ERS-C (SE = 0.022, 95% CI −0.045, 0.045). Based on parent outcome reports, no significant (in)direct associations were found.

#### Examining Attachment Avoidance toward Father

Figure 2D shows that the direct relationship between Attachment Avoidance toward Father and Externalizing problems was not significant (SE = 0.049, *t* = 1.098, *p* = 0.275). Relationship between Attachment Avoidance toward Father and M.ERS-C was significant (SE = 0.167, *t* = 3.314, *p* = 0.001). Relationship between M.ERS-C and Externalizing problems was not significant (SE = 0.029, *t* = −0.016, *p* = 0.987). Examining the CI, no significant associative pathway was supported between Attachment Avoidance toward Father on Externalizing problems via M.ERS-C (SE = 0.015, 95% CI −0.030, 0.032); this is considered borderline and must be interpreted with caution. Based on parent outcome reports, no significant direct associations were found. However, for mother outcome reports, an association via an associative pathway was supported. Father reports did not yield significant associative pathways.

### Parental maladaptive ERS and internalizing problems, associated through associative pathways involving child's maladaptive ERS

#### Examining maternal maladaptive ERS

Figure 3A shows the direct relationship between Maladaptive ERS Mother (M.ERS-M) and Internalizing problems was not significant (SE = 0.018, *t* = 1.552, *p* = 0.125). Relationship between M.ERS-M and M.ERS-C was not significant (SE = 0.077, *t* = 1.465, *p* = 0.147). Relationship between M.ERS-C and Internalizing problems was significant (SE = 0.026, *t* = 4.400, *p* = 0.000). Examining the CI, no significant associative pathway was supported between M.ERS-M on Internalizing problems via M.ERS-C (SE = 0.011, 95% CI −0.006, 0.035). Based on mother outcome reports, (in)direct associations were not significant.

#### Examining paternal maladaptive ERS

Figure 3B shows the direct relationship between Maladaptive ERS Father (M.ERS-F) and Internalizing problems was not significant (SE = 0.021, *t* = 1.899, *p* = 0.062). Relationship between M.ERS-F and M.ERS-C was not significant (SE = 0.083, *t* = 1.644, *p* = 0.105). Relationship between M.ERS-C and Internalizing problems was significant (SE = 0.031, *t* = 4.300, *p* = 0.000). Examining the CI, no significant associative pathway was supported between M.ERS-F on Internalizing problems via M.ERS-C (SE = 0.010, 95% CI −0.002, 0.040); this is considered borderline and must be interpreted with caution. On the contrary, based on father outcome reports, direct associations and associations via an associative pathway were both significant.

### Parental maladaptive ERS and externalizing problems, associated through associative pathways involving child's maladaptive ERS

#### Examining maternal maladaptive ERS

Figure 3C shows the direct relationship between M.ERS-M and Externalizing problems was not significant (SE = 0.023, *t* = 1.242, *p* = 0.218). Relationship between M.ERS-M and M.ERS-C was not significant (SE = 0.080, *t* = 1.232, *p* = 0.222). Relationship between M.ERS-C and Externalizing problems was not significant (SE = 0.033, *t* = −0.086, *p* = 0.932). Examining the CI, no significant associative pathway was supported between M.ERS-M on Externalizing problems via M.ERS-C (SE = 0.004, 95% CI −0.009, 0.008). Based on mother outcome reports, (in)direct associations were not significant.

#### Examining paternal maladaptive ERS

Figure 3D shows the direct relationship between M.ERS-F and Externalizing problems was not significant (SE = .025, *t* = 1.521, *p* = .133). Relationship between M.ERS-F and M.ERS-C was not significant (SE = 0.082, *t* = 1.376, *p* = 0.173). Relationship between M.ERS-C and Externalizing problems was not significant (SE = 0.037, *t* = 0.405, *p* = 0.687). Examining the CI, no significant associative pathway was supported between M.ERS-F on Externalizing problems via M.ERS-C (SE = 0.004, 95% CI −0.007, 0.010). On the contrary, based on father outcome reports, only the significant direct association was found, the indirect associative pathway was not significant.

## Discussion

This study contributes to examining ER as a transdiagnostic factor in CHD populations using multi-informant perspectives (14, 46). The present cross-sectional study examined the extent to which (1) insecure attachment and (2) parental maladaptive ERS are associated with children's psychosocial functioning, both directly and indirectly via associative pathways.

Based on theoretical assumptions about family functioning (23), we hypothesized that insecure attachment and parental maladaptive ERS would be related to internalizing/externalizing problems directly and through children's ERS. While our explorative cross-sectional design precludes examination of ER developmental trajectories, we situate our findings within established developmental frameworks to contextualize observed associations.

## Insecure attachment and children's psychosocial functioning

As hypothesized, Attachment Avoidance toward Mother had a direct effect on self-reported internalizing problems in children with CHD. Contrary to hypothesis, children's self-reports showed that Attachment Anxiety toward Mother/Father and Attachment Avoidance toward Father were not directly associated with self-reported internalizing problems.

Consistent with Morris et al. (23), children's use of maladaptive ERS was part of the pathway associating the three other insecure attachment dimensions (Attachment Anxiety in Mother and Attachment Anxiety/Avoidance in Father) with internalizing problems. The analyses revealed associations via associative pathways without corresponding direct effects (indirect-only pathway).

Several of these associations via associative pathways showed borderline significance and require cautious interpretation (23, 47). Nevertheless, similar findings exist in literature. Brenning and colleagues (62) found evidence for maladaptive ER as a pathway associating insecure attachment and depressive symptoms in youngsters. A meta-analysis also confirmed that maladaptive ER consistently accounts for pathways between insecure attachment and internalizing problems across healthy clinical and non-clinical samples (17). In our CHD sample, children's maladaptive ERS similarly contributed to pathways associating insecure attachment to internalizing symptoms, with effect sizes (CSIE) that were small but statistically reliable. In CHD, these pathways operate within a unique psychological context with bimodal peaks in early childhood and adolescence (14). High stress from cumulative medical adversity may amplify attachment-ER associations.

Multi-informant findings revealed crucial differences. Parent outcome reports showed no direct effects between insecure attachment and children's internalizing problems. Child self-reports and mother outcome reports showed no associative pathway from Attachment Avoidance toward Mother on internalizing problems. However, father outcome reports did reveal an association via associative pathway (CI values close to zero). This shows that informant differences may reflect family dynamics distinctions. Fathers may perceive or report children's behavior differently due to varying roles in daily caregiving or emotional involvement (48). Further research should include family contextual variables (e.g., coresidency, custody, parental conflict) as potential moderators to clarify differences in internalizing problems among youngsters with CHD (49).

For externalizing problems, we confirmed the hypothesis of a direct association between both insecure attachment dimensions toward mothers and children's self-reported externalizing problems. However, no evidence was found for direct effects between insecure attachment toward fathers and externalizing problems, nor for associations via associative pathways via children's maladaptive ERS. Mother outcome reports partially confirmed these findings by showing a direct effect of Attachment Anxiety (but not Attachment Avoidance) toward Mother. Moreover, mother reports, unlike child or father reports, showed a borderline associative pathway (CI close to zero and interpreted cautiously) from Attachment Avoidance toward Father, on externalizing problems.

Insecure attachment and children's maladaptive ERS play distinct roles in internalizing vs. externalizing problems. This aligns with literature showing heterogeneous CHD psychosocial outcomes shaped by medical, neurodevelopmental, and family factors (16, 50, 51). Our study extends this work by identifying family-level mechanisms that may underlie this variability.

Attachment toward mothers plays a different role compared to attachment toward fathers. Our findings reveal stronger and more consistent maternal effects that can be understood through several interconnected factors. First, systematic differences in parental caregiving roles likely contribute to these patterns (52–55). Mothers typically assume primary caregiving responsibilities and experience higher stress, while fathers often adopt more supportive secondary roles (56, 57). In CHD families, this dynamic intersects with systemic illness processes: Delaney (2022) noted that the “shared experience” of illness can strain marriages and lead parents to become emotionally closed off (58). Parents may influence their children's ER development differently (53, 54), with mothers' primary caregiving role potentially creating stronger pathways between maternal attachment security and children's regulatory capacities. A broad age range further shapes these patterns. Children in middle childhood and adolescence maintain stronger ties with primary caregivers while gradually gaining autonomy from secondary caregivers (59), potentially magnifying maternal attachment influences on children's ER. Next, beyond caregiving role differentiation, measurement sensitivity factors may contribute to stronger maternal effects. While parental reports supported several child self-report findings, informant discrepancies appeared. These differences may reflect genuine variability in perspective, role distribution, and emotional attunement rather than measurement error. Each informant may access different relational or behavioral aspects of the child's functioning. Mothers may be more attuned to daily behavioral cues and regulatory struggles, while fathers observe different contexts or developmental domains.

Finally, sample-related limitations may have affected our ability to detect paternal effects. Research on fathers in pediatric populations remains limited (55), and cross-sectional design constraints may obscure the temporal development of father–child attachment-ER pathways. These methodological considerations, combined with caregiving role differences and measurement sensitivities collectively suggest that maternal attachment effects may appear more robust due to structural, methodological, and sample-specific factors, though this should not diminish the potential importance of father–child relationships in CHD families.

## Parental maladaptive ERS and Children's psychosocial functioning

Contrary to hypotheses, our data based on child and mother outcome reports showed that parents' maladaptive ERS were not directly associated with internalizing or externalizing problems in the children with CHD. However, data based on father reports showed that fathers' maladaptive ERS were directly associated with both internalizing and externalizing problems in children with CHD. These findings align with the literature (60), which shows that parental maladaptive ERS are significantly associated with internalizing problems but not with externalizing problems in healthy children (61).

No associative pathways were discovered between parental maladaptive ERS and children's psychosocial functioning, except for a borderline association through child ERS on internalizing problems according to father reports (CIs close to zero and interpreted cautiously).

Several findings call for discussion. Although theoretical models emphasize intergenerational transmission of maladaptive ERS, we found no significant parent–child associations (path a, Figure 3) (53, 54). This discrepancy may reflect limitations of our cross-sectional design, which cannot capture developmental ER processes over time, or the reliance on parental self-reports that overlook parenting practices and relationship quality (23). Overall, parents' maladaptive ERS were not consistently associated with children's maladaptive ER. In general, prior work shows that parents who use more maladaptive ERS have children who also use more maladaptive ERS and exhibit more internalizing symptoms (60, 62, 63), for externalizing behaviors the evidence is less clear (61). In CHD families, overprotective parenting may further constrain observational learning, as children are often treated as “childlike” and shielded from parental coping behaviors (58).

## Study strengths and limitations

This study has several notable strengths. It includes a large participant sample, encompassing patients and assessments from both parents. The multi-informant approach examined attachment, ER, and psychosocial functioning from both parental and child perspectives. The patients had a wide variety of mild to complex CHD pathologies, representing the typical CHD population. The final sample exceeded the minimum required size based on a priori power analysis, enhancing statistical robustness. Although O’Connor et al. (15) showed lower psychosocial functioning in moderate-severe CHD (15), our findings suggest that family-level mechanisms also contribute beyond medical severity.

Some limitations should be acknowledged. First, the cross-sectional design precludes conclusions about temporal or causal relationships, particularly when it comes to associative pathway analyses, which inherently assume temporal precedence. Importantly, the design also prevents formal modeling of ER developmental trajectories. This limits our ability to examine how ER skills evolve over time in children with CHD. Future research should replicate these findings longitudinally, incorporate developmental modeling to better capture ER processes, and systematically examine how CHD severity interacts with family-level and emotional mechanisms. Prospective studies stratified by CHD complexity could help clarify whether these associative pathways apply equally across severity levels, or whether greater illness burden heightens vulnerability to attachment and ER effects.

Second, several factors may limit generalizability. Non-response bias cannot be ruled out, as the study design did not allow comparison of psychosocial characteristics between respondents and non-respondents. Although our sample was clinically representative, reflecting the distribution of pathologies and procedures in our complete surgical database, vulnerable subgroups may still be underrepresented. Due to the study design, we excluded children with intellectual disabilities or genetic syndromes, and families with language barriers creating a potential ethnic bias. Moreover, voluntary participation could also create a selection bias as it could not be ruled out that the most vulnerable families did not participate. This could have led to underestimation of psychosocial difficulties. The absence of a healthy control group limits interpretability of symptom levels compared to typical developmental trajectories.

It was beyond the scope of this research to study the impact of all clinical parameters that might have had an influence on neurodevelopmental outcome (e.g., cyanosis, bypass duration, ICU length of stay). Also parental age, socioeconomic status, and children's language development were not assessed, though they may influence psychosocial outcomes in CHD (64–66).

Variability in family participation and assessment methods may limit the generalizability of our findings. Specifically, we were unable to recruit both parents for each child, which restricts direct mother–father comparisons. Relying solely on questionnaire-based reports also limits insights into underlying emotional mechanisms.

Finally, the COVID-19 pandemic, with its associated stressors like heightened parental stress, disruption of children's routines, and limited healthcare access for families with CHD, affected findings. Studies showed that youngsters with CHD, particularly those with complex conditions, showed reduced resilience compared to healthy peers (67). Because data collection coincided with this pandemic, our findings may partly capture family functioning under exceptional stress conditions rather than typical circumstances. As such, they should be interpreted with caution and may not fully generalize to CHD families outside of this unique context.

## Future directions and clinical implications

Future research should focus on further understanding the role of emotional processing in explaining psychosocial functioning, resilience, and wellbeing in children with CHD (49). Key priorities include longitudinal studies examining developmental timing (14) and CHD complexity (15) as moderators. For instance, maladaptive ERS may play a stronger role in families of children with severe CHD, where illness burden and neurodevelopmental vulnerabilities are greatest. It will also be important to examine interaction terms (e.g., between biological sex and ER). Quantitative, qualitative, and observational studies with healthy controls are needed to contextualize symptom levels. Future research should explore adaptive ERS alongside maladaptive strategies to provide insight into the full spectrum of ER within this population. Secure attachment and adaptive ERS represent the protective counterpart to the risk processes examined here, underscoring their clinical relevance for developing comprehensive interventions. In addition, characterizing non-respondents will help ensure that at-risk groups are more accurately represented, while incorporating measures of parental psychopathology will offer a broader understanding of family dynamics. Understanding these emotional processes is crucial for implementing effective interventions for both children and their families.

Based on our findings, practitioners should consider the following suggestions. Given its chronic nature, CHD care requires transition from an acute to a long-term holistic multi-disciplinary approach. Routine monitoring of ER and psychosocial functioning is recommended. Addressing children's mental health around surgery is important to mitigate distress and prevent psychosocial issues during development. Psychosocial interventions, effective in other chronic illness groups (68), should be tailored to both family and individual levels for optimized effectiveness. Family-centered developmental care should promote parent–child bonding, emotional expression, parenting skills, and problem solving (51, 69). These findings reinforce the clinical value of incorporating both mother and father perspectives, where possible, into psychosocial screenings. A child- and family-centered model should consider differences in parental perception and involvement to tailor interventions accordingly. Clinical practice should focus on strengthening parent–child relationships, providing ER training for children, and offering emotional support for parents. This approach acknowledges the importance of mitigating risk factors and fostering protective processes, aiming for improved psychosocial outcomes and resilience.

## Conclusion

Children's self-reports revealed that insecure attachment to the mother, but not the father, was directly related to their psychological difficulties, particularly internalizing symptoms. These effects were partially associated through associative pathways involving the child's maladaptive ERS. Mother-reported outcomes confirmed these associations, while father reports revealed associations via associative pathways. Contrary to our expectations, neither child nor mother reports revealed significant associations between parental maladaptive ERS and child outcomes. In contrast, father-reported outcomes showed both direct effects of their own maladaptive ERS on the child's psychological difficulties, including internalizing problems, as well as associations through associative pathways involving the child's ERS. Together, these results suggest that insecure attachment and child ERS primarily are associated with internalizing symptoms, while externalizing symptoms were more selectively associated with maternal attachment. Findings varied by informant, underscoring the importance of using a multi-informant approach. Clinically, the findings emphasize the need for targeted interventions that strengthen secure parent–child bonds and enhance children's ER capacity. Future research should include fathers, observational designs, and longitudinal approaches to better understand these mechanisms.

## Data Availability

The datasets generated and/or analyzed for this study will be available on reasonable request from the corresponding author.
