# Supplementary material for: Psychosocial functioning in children with a congenital heart disease: attachment and emotion regulation strategies of children and parents as explanatory factors
Source: Front Cardiovasc Med. 2025 Dec 17;12:1658513. doi: 10.3389/fcvm.2025.1658513 (PMC12754529; doi:10.3389/fcvm.2025.1658513)
Supplement: Supplementary file 1 [file Datasheet1.pdf]

Supplementary Table S1. *Informant response patterns across participating families (N = 218)*

**Respondent constellation***	**Number of families (N)**	**Percentage (%)**
Child + Mother + Father (complete triad)	92	42.2
Child + Mother only	42	19.3
Child + Father only	10	4.6
Mother + Father only (no child report)	41	18.8
Mother only	17	7.8
Father only	10	4.6
Child only	6	2.7
**Total families**	**218**	**100**

Note. N = number. *The total number of unique participating families was 218. Research questions were examined using a multi-informant approach. This included child self-report (*N* = 107 for the SDQ, completed by children aged 11-18 years; *N* = 150 for the ECR-RC and FEEL-KJ, completed by children aged 8-18 years), and mother-reported (*N* = 192) and father-reported (*N* = 153) outcome measures. The difference in the number of participating children for the questionnaires is due to the SDQ being suitable only for children aged 11 and above, resulting in a smaller subgroup for this specific measure. This table outlines the number and percentage of families for which data were available from one, two, or all three informants (i.e., child, mother, father). Participation was voluntary and not all families submitted data from all members. Nonetheless, all available data were retained and included in the analyses using appropriate statistical handling of missingness.

Supplementary Table S2. *Descriptives and Pearson correlations among mother study variables* (*N* = 192).

		M	*(SD)*	1	2	3	4	5	6	7	8
1	Att. Anx. M	9.56	(5.08)	-							
2	Att. Av. M	18.55	(8.71)	.326**	-						
3	Att. Anx. F	9.27	(5.31)	.679**	.164	-					
4	Att. Av. F	21.00	(8.17)	.190*	.602**	.349**	-				
5	Mal. ERS M	92.52	(17.70)	.072	.239**	.125	.215*	-			
6	Mal. ERS C	80.93	(14.22)	.401**	.264	.389**	.333**	.156	-		
7	Int.	5.19	(3.83)	.136	.054	.239**	.166	.080	.335**	-	
8	Ext.	5.19	(4.00)	.233**	.018	.144	-.076	.004	.036	.501**	-

Note. Att. Ax. M = Attachment Anxiety towards Mother*; Att. Av. M* = Attachment Avoidance towards Mother*; Att. Ax. F* = Attachment Anxiety towards Father*; Att. Av. F* = Attachment Avoidance towards Father*; Mal. ERS M (tot)* = Mother's maladaptive Emotion Regulation Strategies (Total); *Mal. ERS C* = Child's maladaptive Emotion Regulation Strategies (Total); *Int.* = Internalizing problems (mother-reports); *Ext.* = Externalizing problems (mother-reports); *M* = Mean; *SD* = Standard Deviation; *$p < .05$ (2-tailed), **$p < .01$ (2-tailed).

Supplementary Table S3. *Descriptives and Pearson correlations among father study variables* (*N* = 153).

		M	*(SD)*	1	2	3	4	5	6	7	8
1	Att. Anx. M	9.56	(5.08)	-							
2	Att. Av. M	18.55	(8.71)	.326**	-						
3	Att. Anx. F	9.27	(5.31)	.679**	.164	-					
4	Att. Av. F	21.00	(8.17)	.190*	.602**	.349**	-				
5	Mal. ERS F	12.87	(4.41)	.246*	.108	.340**	.104	-			
6	Mal. ERS C	80.93	(14.22)	.401**	.264**	.389**	.333**	.406**	-		
7	Int.	4.44	(3.65)	.182*	.057	.233*	.188*	.352**	.365**	-	
8	Ext.	5.22	(3.86)	.264**	-.034	.150	-.051	.217*	.091	.491**	-

Note. Att. Ax. M = Attachment Anxiety towards Mother*; Att. Av. M* = Attachment Avoidance towards Mother*; Att. Ax. F* = Attachment Anxiety towards Father*; Att. Av. F* = Attachment Avoidance towards Father*; Mal. ERS F (tot)* = Father's maladaptive Emotion Regulation Strategies (Total); *Mal. ERS C* = Child's maladaptive Emotion Regulation Strategies (Total); *Int.* = Internalizing problems (father-reports); *Ext.* = Externalizing problems (father-reports); *M* = Mean; *SD* = Standard Deviation; *$p < .05$ (2-tailed), **$p < .01$ (2-tailed).

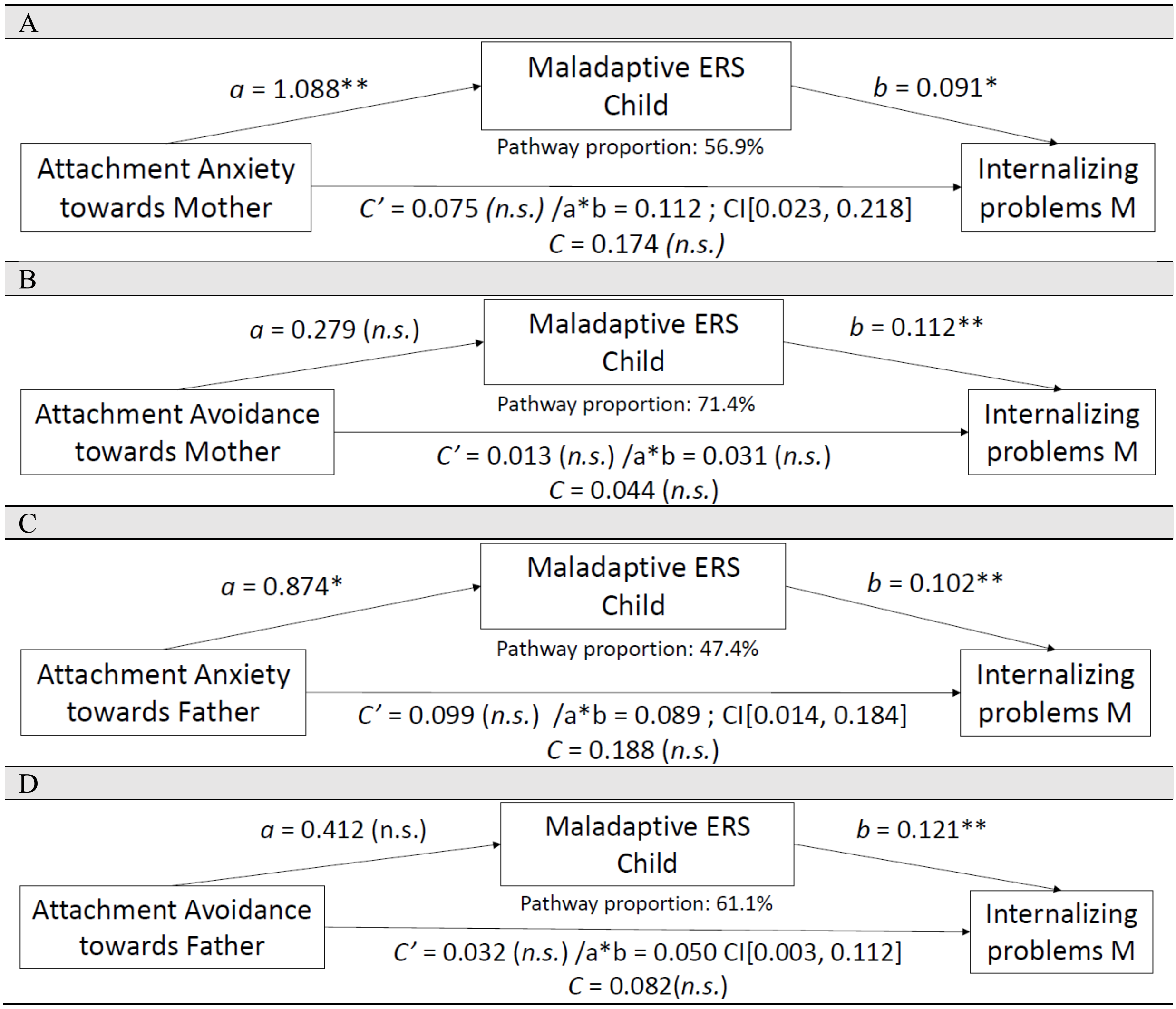

Supplementary Figure S4. Associations between (A) Attachment Anxiety towards Mother (*N* = 114), or (B) Attachment Avoidance towards Mother (*N* = 113), or (C) Attachment Anxiety towards Father (*N* = 112), or (D) Attachment Avoidance towards Father (*N* = 112), Maladaptive Emotion Regulation Strategies Child and Internalizing problems in 8 to 18-year old children with CHD (M = mother outcome reports). Controlled for the child's education level, birth order and family composition in these associative pathway analyses. *n.s.* = not significant; Bonferroni correction (p-value of .05 divided by 4 = .013): $*p \leq .013$, $**p \leq .001$. *N* refers to the effective sample size per associative pathway model.

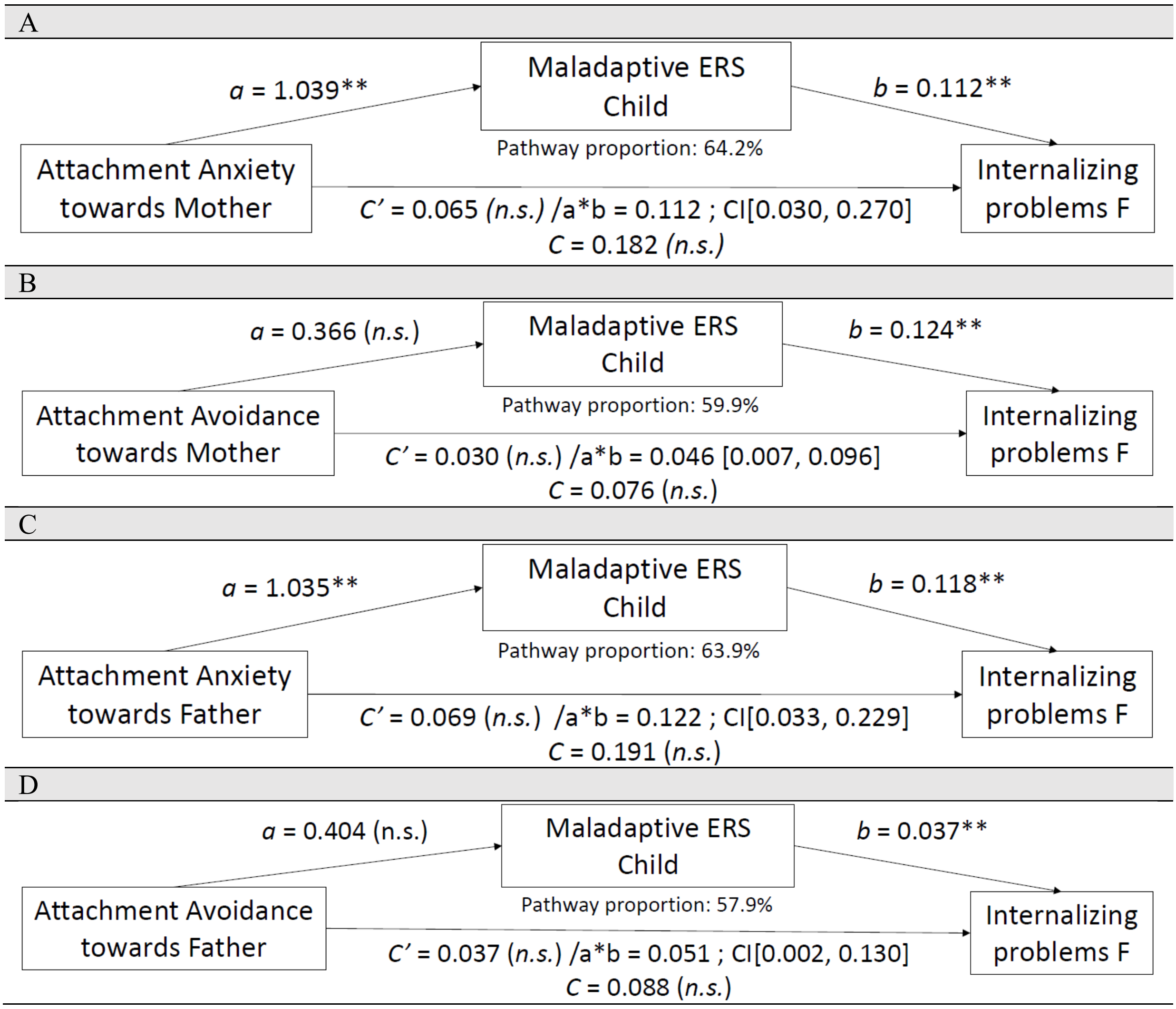

Supplementary Figure S5. Associations between (A) Attachment Anxiety towards Mother (*N* = 97), or (B) Attachment Avoidance towards Mother (*N* = 97), or (C) Attachment Anxiety towards Father (*N* = 99), or (D) Attachment Avoidance towards Father (*N* = 99), Maladaptive Emotion Regulation Strategies Child and Internalizing problems in 8 to 18-year old children with CHD (F = father outcome reports). Controlled for the child's education level and number of surgeries in these associative pathway analyses. *n.s.* = not significant; Bonferroni correction (*p*-value of .05 divided by 4 = .013): $*p \leq .013$, $**p \leq .001$. *N* refers to the effective sample size per associative pathway model.

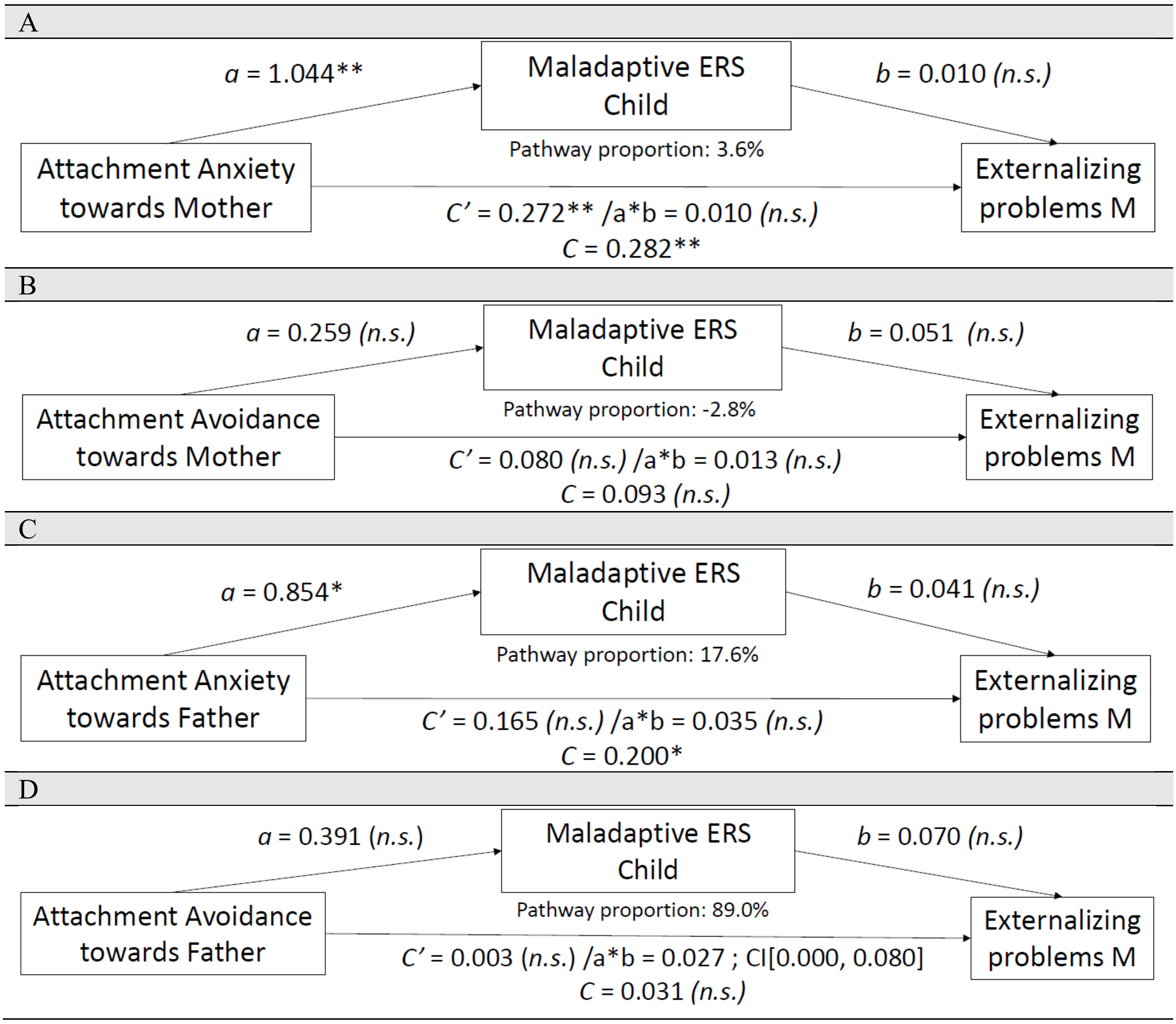

Supplementary Figure S6. Associations between (A) Attachment Anxiety towards Mother (*N* = 116), or (B) Attachment Avoidance towards Mother (*N* = 115), or (C) Attachment Anxiety towards Father (*N* = 114), or (D) Attachment Avoidance towards Father (*N* = 114), Maladaptive Emotion Regulation Strategies Child and Externalizing problems in 8 to 18-year old children with CHD (M = mother outcome reports). Controlled for the child's education level, the child's age and the child's biological sex in these associative pathway analyses. *n.s.* = not significant; Bonferroni correction (p-value of .05 divided by 4 =.013): $^{*}p \leq .013$, $^{**}p \leq .001$. N refers to the effective sample size per associative pathway model.

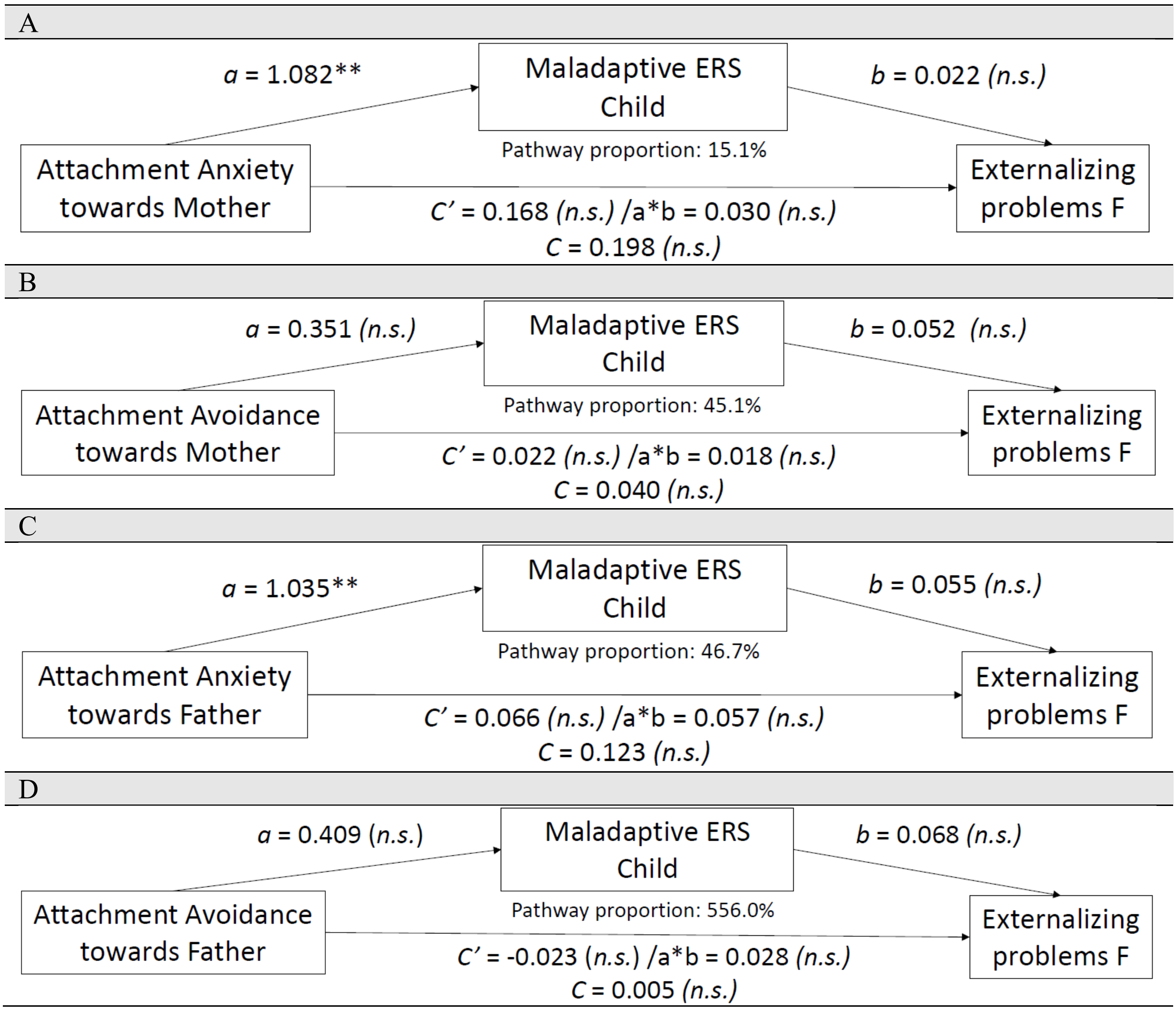

Supplementary Figure S7. Associations between (A) Attachment Anxiety towards Mother (N = 97), or (B) Attachment Avoidance towards Mother (N = 97), or (C) Attachment Anxiety towards Father (N = 99), or (D) Attachment Avoidance towards Father (N = 99), Maladaptive Emotion Regulation Strategies Child and Externalizing problems in 8 to 18-year old children with CHD (F = father outcome reports). Controlled for the child's education level, the child's age, the number of surgeries and the timing of diagnosis in these associative pathway analyses. *n.s.* = not significant; Bonferroni correction (p-value of .05 divided by 4 =.013): *$p \leq .013$, **$p \leq .001$. N refers to the effective sample size per associative pathway model.

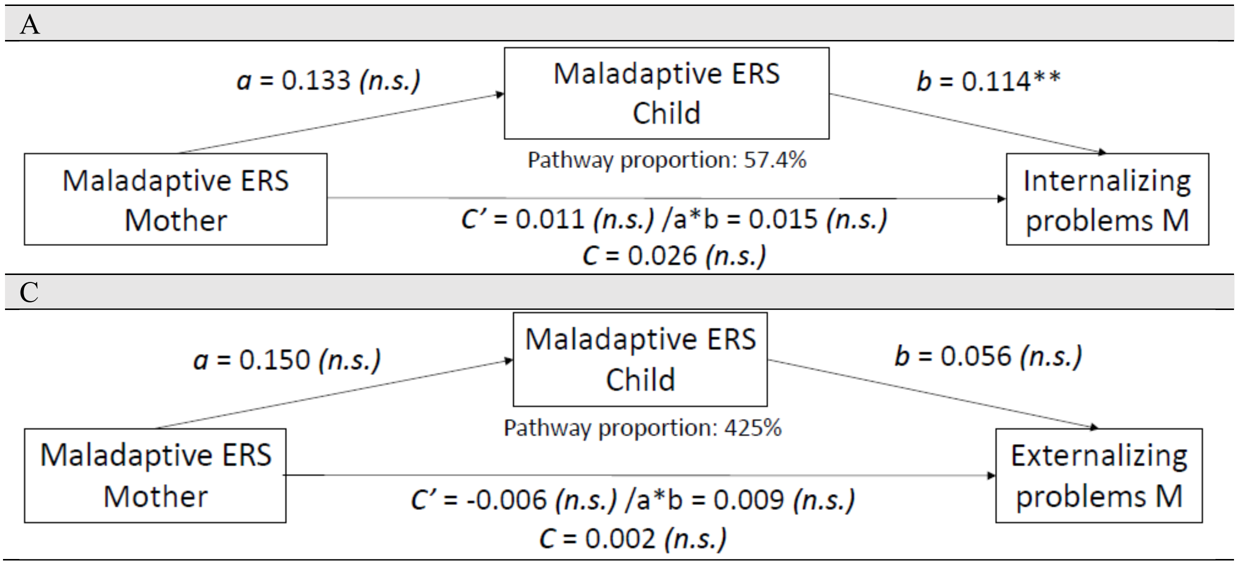

Supplementary Figure S8. Associations between (A) Mother's Maladaptive Emotion Regulation Strategies (N = 110), Maladaptive Emotion Regulation Strategies Child and Internalizing problems in 8 to 18-year old children with CHD (M = mother outcome reports) or associations between (C) Mother's Maladaptive Emotion Regulation Strategies (N = 112), Maladaptive Emotion Regulation Strategies Child and Externalizing problems in 8 to 18-year old children with CHD (M = mother outcome reports). Controlled for the child's education level, birth order and family composition in these associative pathway analyses when 'Internalizing problems' was the outcome variable. Controlled for the child's education level, the child's age and the child's biological sex in these associative pathway analyses when 'Externalizing problems' was the outcome variable. *n.s.* = not significant; Bonferroni correction (p-value of .05 divided by 2 =.025): *$p \leq .025$, **$p \leq .001$. N refers to the effective sample size per associative pathway model.

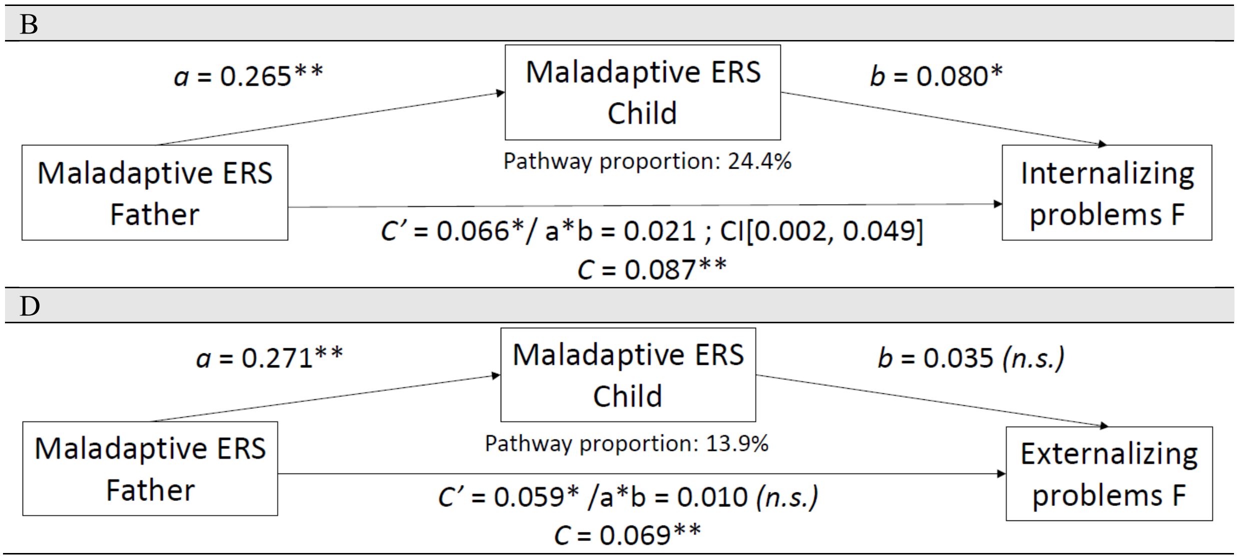

Supplementary Figure S9. Associations between (B) Father's Maladaptive Emotion Regulation Strategies (N = 94), Maladaptive Emotion Regulation Strategies Child and Internalizing problems in 8 to 17-year old children with CHD (F = father outcome reports), or associations between (D) Father's Maladaptive Emotion Regulation Strategies (N = 94), Maladaptive Emotion Regulation Strategies Child and Externalizing problems in 8 to 18-year old children with CHD (F = father outcome reports). Controlled for the child's education level and number of surgeries when 'Internalizing problems' was the outcome variable. Controlled for the child's education level, the child's age, the number of surgeries and the timing of diagnosis in these associative pathway analyses when 'Externalizing problems' was the outcome variable. *n.s.* = not significant; Bonferroni correction (p-value of .05 divided by 2 =.025): *$p \leq .025$, **$p \leq .001$. N refers to the effective sample size per associative pathway model.
